# Presynaptic Membrane Receptors Modulate ACh Release, Axonal Competition and Synapse Elimination during Neuromuscular Junction Development

**DOI:** 10.3389/fnmol.2017.00132

**Published:** 2017-05-16

**Authors:** Josep Tomàs, Neus Garcia, Maria A. Lanuza, Manel M. Santafé, Marta Tomàs, Laura Nadal, Erica Hurtado, Anna Simó, Víctor Cilleros

**Affiliations:** Unitat d’Histologia i Neurobiologia (UHN), Facultat de Medicina i Ciències de la Salut, Universitat Rovira i VirgiliReus, Spain

**Keywords:** postnatal synapse elimination, axonal competition, acetylcholine release, voltage-dependent calcium channels, muscarinic acetylcholine receptors, protein kinases, TrkB, PKC

## Abstract

During the histogenesis of the nervous system a lush production of neurons, which establish an excessive number of synapses, is followed by a drop in both neurons and synaptic contacts as maturation proceeds. Hebbian competition between axons with different activities leads to the loss of roughly half of the neurons initially produced so connectivity is refined and specificity gained. The skeletal muscle fibers in the newborn neuromuscular junction (NMJ) are polyinnervated but by the end of the competition, 2 weeks later, the NMJ are innervated by only one axon. This peripheral synapse has long been used as a convenient model for synapse development. In the last few years, we have studied transmitter release and the local involvement of the presynaptic muscarinic acetylcholine autoreceptors (mAChR), adenosine autoreceptors (AR) and trophic factor receptors (TFR, for neurotrophins and trophic cytokines) during the development of NMJ and in the adult. This review article brings together previously published data and proposes a molecular background for developmental axonal competition and loss. At the end of the first week postnatal, these receptors modulate transmitter release in the various nerve terminals on polyinnervated NMJ and contribute to axonal competition and synapse elimination.

## Introduction

“During the development of the nervous system there is an initial overproduction of synapses” (Lanuza et al., 2014) that promotes wide-ranging connectivity and which is followed by an activity-dependent reduction in their number (Thompson, 1985; Bourgeois and Rakic, 1993). “This refines connectivity and increases specificity” (Nadal et al., 2016). Hebbian competition between nerve endings with different activities (the less active result eliminated) is the fundamental feature of the process which leads to the elimination of half of the contacts produced and the strengthening of the remaining contacts (Fields and Nelson, 1992; Sanes and Lichtman, 1999; Zorumski and Mennerick, 2000). Synaptic contacts are lost throughout the nervous tissues during histogenesis (Bourgeois and Rakic, 1993). In the visual system, thalamic axons detach from cortical cells (Hubel et al., 1977; Huberman, 2007); in the cerebellum, climbing fibers (CFs) disconnect from Purkinje cells (PC; Daniel et al., 1992; Hashimoto and Kano, 2005); in autonomic ganglia, axonal inputs disconnect from ganglionar neurons (Lichtman, 1977); and at the neuromuscular junction (NMJ), motor nerve endings disconnect from muscle cells (Benoit and Changeux, 1975; O’Brien et al., 1978). In some neural circuits a given presynaptic axon type innervates only one postsynaptic cell at the end of the competition process (i.e., only one climbing axon persists over the dendritic arbor of a PC in the cerebellar cortex). However, most neurons were polyinnervated by various axons in the adult and the mechanism of axonal competition and selection of some nerve endings was even more sophisticated.

In newborn animals, skeletal muscle fibers are polyinnervated in the NMJ by several motor axons (Redfern, 1970; Brown et al., 1976; Ribchester and Barry, 1994), but at the end of the competitive interactions between the nerve endings, endplates are innervated by a solitary axon (Benoit and Changeux, 1975; O’Brien et al., 1978; Jansen and Fladby, 1990; Sanes and Lichtman, 1999). This peripheral synapse has long been used as a paradigm for studying the principles of synapse development and function (Keller-Peck et al., 2001; Lanuza et al., 2002; Santafé et al., 2009a; Garcia et al., 2011; Lichtman and Tapia, 2013). There is evidence to suggest that several presynaptic receptors (muscarinic acetylcholine autoreceptors (mAChR), adenosine autoreceptors (AR) and tropomyosin-related kinase B receptor (TrkB)) play an important role by allowing the nerve terminals to communicate in the competition that leads to synapse loss in the NMJ (Santafé et al., 2006; Amaral and Pozzo-Miller, 2012; Nadal et al., 2016). The mAChR may be of particular importance. The involvement of mAChR in the elimination process “may allow direct competitive interaction between nerve endings through differential activity-dependent acetylcholine (ACh) release. The more active endings may directly punish the less active ones or reward themselves” (Nadal et al., 2016). Differences in the amount of activity of the terminal axons in competition but also their timing is important. Asynchronous activity promotes synapse elimination whereas synchronous activity prevents it (Favero et al., 2012). Our results indicate that the weakest nerve endings (those that evoke endplate potentials (EPP) with the least quantal content in dual junctions) have an ACh release inhibition mechanism, based on muscarinic autoreceptors and coupled to protein kinase C (PKC) and voltage-dependent calcium channels (VDCC), that can depress the ACh release capacity in these endings and even contribute to functionally disconnect the synapse (Santafé et al., 2003, 2004, 2007a, 2009a,b; Tomàs et al., 2011). We suggest that this muscarinic “mechanism plays a central role in the elimination of redundant neonatal synapses because functional axonal withdrawal can indeed be temporarily reversed by mAChR, VDCC or PKC block” (Santafé et al., 2007b, 2009a; Tomàs et al., 2011). In addition, M_1_, M_2_ and M_4_ muscarinic subtypes cooperate to favor axonal competition at the end of the first postnatal week and promote the full sequence of axonal loss and synapse elimination shortly thereafter (Nadal et al., 2016).

However, different local effectivenesses and motorneuron activities are key to eventual success or failure, since an axon that fails at one muscle cell can win the competition at another (Keller-Peck et al., 2001), which suggests the participation of other signaling pathways and possible postsynaptic muscle cell-derived factors. Our results suggest that TrkB receptor-mediated, brain derived neurotrophic factor (BDNF) signaling plays this role and cooperates with muscarinic signaling (Tomàs et al., 2011; Nadal et al., 2016, 2017b).

This review article collects and reevaluates previously published data on the local involvement of the presynaptic mAChR and TrkB pathway in ACh release and axonal elimination. We propose a molecular background for developmental axonal loss.

## Experimental Conditions

We attempted to characterize the funcional capacity of the various motor axons that are in competition at the polyinnervated NMJ. “The homogeneity of the experimental conditions needs to be carefully defined in a review study such as this one” (Tomàs et al., 2014). For the electrophysological experiments, only *ex vivo* *Levator auris longus* (LAL) muscles from P6-P7 mice (Swiss mice) or rat (Sprague-Dawley) were studied and the basic procedures have been extensively described (Santafé et al., 2003, 2004, 2009a; Tomàs et al., 2011). Briefly, to prevent stimulation-induced contractions, neonatal muscles were paralyzed with μ-CgTX-GIIB or occasionally cut on either side of the main intramuscular nerve branch. “The nerve was stimulated with increasing intensity from zero until an EPP was observed. If the size and latency of the EPP remained constant as the stimulus was increased, we concluded that the endplate was mono-innervated (*M* endings). In endplates with polyneuronal innervation, increasing the stimulus amplitude caused one or more axons to be recruited, which produced a stepwise increment in the EPP” (Redfern, 1970). Specifically, “with dually innervated fibers (the most affordable polyinnervation condition), a second EPP can appear after the first one when the intensity of the electrical stimulus is increased. This compound EPP is built by recruiting two axons. We calculated the EPP amplitude of the second axon response by subtracting the first EPP amplitude from the compound EPP” (Garcia et al., 2010b). Usually, these EPPs have different amplitudes because “the size of an EPP is not related to the threshold of the axon” (Santafé et al., 2009a) that produces it. “We refer to the axon terminals that produce these synaptic potentials as the weak (*W*, smallest EPP) and strong (*S*, largest EPP) nerve endings (and their synapses)”. In addition, we observed (Santafé et al., 2009a; Tomàs et al., 2011) that some nerve terminals go silent (do not evoke EPP on stimulation) before they completely retract and before the end of the functional elimination period, “but retain certain capabilities for evoked release that can be pharmacologically recovered (*R*, recovered endings)” (Tomàs et al., 2011). In polyinnervated synapses, “quantal responses clearly decrease in both size and number before axonal withdrawal is completed (Dunia and Herrera, 1993; Colman et al., 1997). Neurotransmitter release from the axon that survives is characterized by a greater quantal content, whereas the efficiency of the input(s) removed decreases progressively, since a small quantal content is associated with reduced postsynaptic receptor density (Colman et al., 1997; Culican et al., 1998)” (see Santafé et al., 2002). “Imposed changes in synaptic activity can accelerate or delay this developmental synapse elimination process (Jansen and Fladby, 1990), and in most cases, deviations from the normal physiological tempo are for several hours or even days” (see also Nelson, 2005; Tomàs et al., 2011). However, when we studied the *R* endings, we observed a fast response (1 h) of some motor nerve terminals, which recovered ACh release by acute exposure to modulators of certain molecular pathways involved in neurotransmission. “We used intracellular recordings of the evoked synaptic potentials to observe the number of functional inputs for a large number of NMJs. Then we calculated the mean value, defined as the polyinnervation index of the muscle studied (PI)” in control P6-P7 rodent muscles the PI was 1.63 ± 0.14 with a 47.92% ± 2.08 of monoinnervated junctions (Lanuza et al., 2001; Santafé et al., 2001), and finally we studied the “effect on PI of blocking or activating several key molecules involved in ACh release” (Tomàs et al., 2011). A rapid increase in PI can indicate the recruitment of some silent nerve endings that transitorily recover transmission (*R* endings).

In summary, we analyzed how neurotransmission is affected by interfering with muscarinic and neurotrophin signaling in *M, S and W* P7 synaptic contacts on dual junctions, and the possible appearance of silent contacts (*R*) and compared these cases with neurotransmission in the mature adult NMJ (P30; *A* nerve endings; Santafé et al., 2001, 2002, 2004, 2009b; Garcia et al., 2010d; Tomàs et al., 2011).

**Figure 1:**
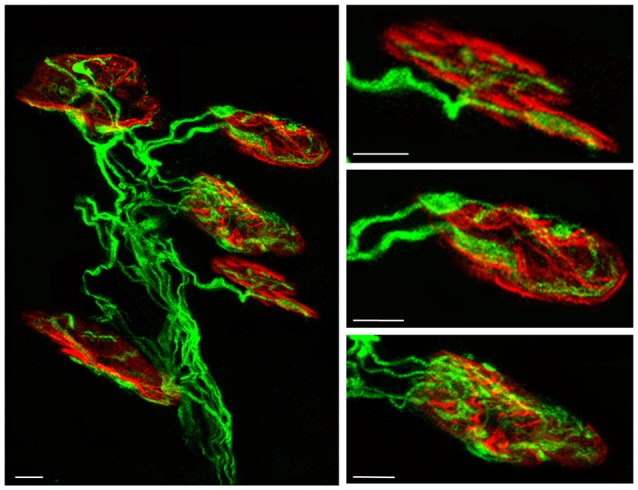
**Confocal immunofluorescence images.** The pictures show representative confocal fluorescence images of monoinnervated and polyinnervated synapses from C57BL/6J P7 control mice. The levator auris longus (LAL) neuromuscular junctions (NMJs) show the axons stained by 200-kD neurofilament antibody in green and the postsynaptic nicotinic acetylcholine receptor (nAChR) clusters stained in red with TRITC-α- BTX. Scale bar: 10 μm.

Finally, we performed direct “axonal counts in confocal LAL preparations (average number of axonal connections per NMJ) from B6.Cg-Tg (Thy1-YFP)16 Jrs/J mice (hereinafter YFP). Transgenic mice express spectral variants of GFP (yellow-YFP) at high levels in motor neurons and axons are brightly fluorescent all the way to the terminals” (Nadal et al., 2016). In most cases, we checked the results with C57BL/6J mice and the axons were shown with an antibody against 200-kD neurofilament protein. LAL muscles were processed to detect the postsynaptic nicotinic ACh receptors (nAChRs) with TRITC-α- BTX (Figure 1). In these histological preparations we counted “the percentage of singly-, dually- and triply- (or more) innervated synapses at P7, P9 and P15 postnatal days with no experimental manipulation (control), and also after two (days 5, 6), four (days 5–8) and 10 (days 5–14) subcutaneous applications of muscarinic and TrkB receptor signaling-related substances” (Nadal et al., 2016; see also Nadal et al., 2017a,b).

## Muscarinic Signaling

### mAChR in the NMJ

There is no consensus about which mAChR subtypes are present in the nerve terminals on the NMJ (Garcia et al., 2005; Wright et al., 2009). In immunohistochemistry assays, most antibodies seemed to detect more than one subtype but in knockout mice their specificity was not clearly determined (Jositsch et al., 2009). Some studies (Wright et al., 2009) only unquestionably observed the M_2_ subtype in the adult nerve endings. In adult and newborn NMJs, we observed the probable presence of M_1_, M_2_, M_3_ and M_4_ subtypes in the cells that construct the synapse (Garcia et al., 2005). In addition, intracellular recording of the synaptic transmission using selective and unselective muscarinic agonists and blockers show that some of these receptors have a regulatory influence on ACh release in developing (Santafé et al., 2001, 2002, 2003, 2004, 2007b, 2009a) and adult synapses (Santafé et al., 2005, 2006, 2007b). Using genetic approaches, it has been observed that motor axon terminals are unstable without M_2._ Some loss of terminal branches occurs in the M_2_ KO mice (Wright et al., 2009). In this context, the neuronal connectivity in the visual cortex was altered by the absence of M_2_/M_4_ mAChR (Groleau et al., 2014).

### mAChR in ACh Release

**Figure 2:**
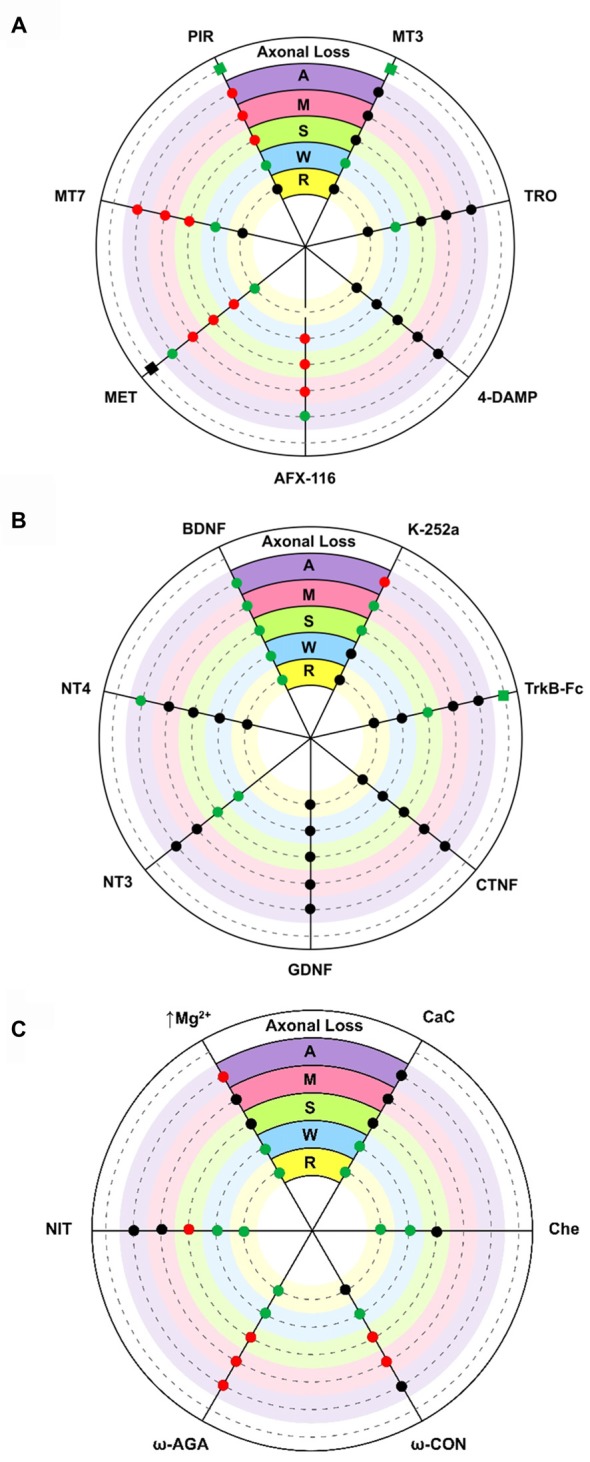
**Diagrams showing an overall representation of the data.** The diagrams show the effect of several substances on acetylcholine (ACh) release (evoke endplate potential, EPP size represented by circles: increase [green], decrease [red] and no change [black] with respect to untreated controls) in developing (P7) single axons on monoinnervated junctions [M], the strong [S] and weak [W] synaptic contacts on dual junctions and in adult (P30) nerve endings [A]. Silent synaptic contacts [R] can be observed in some NMJs in treated muscles after recovering ACh release. Here, R shows the effect of some substances on the polyinnervation index (PI, the mean number of axons per synapse) of these treated muscles (green = increased PI, black = no change). The axonal loss rate (represented with squares in the outermost concentric layer in **A** and **B**) is quantified by direct axonal counts in confocal LAL preparations from B6.Cg-Tg (Thy1-YFP)16 Jrs/J mice. In these histological preparations we counted the percentage of singly-, dually- and triply- (or more) innervated synapses at P7 after 2 days of subcutaneous applications of several muscarinic and TrkB substances. Delayed axon loss in red squares, accelerated loss in green and no change in black. **(A)** shows the effect of several subtype-selective muscarinicsubstances. The M_2_ receptor is selectively blocked with methoctramine (MET) or AFX-116. The M_1_ receptor is selectively blocked with pirenzepine (PIR) or muscarinic toxin 7 (MT7). The M_3_ subtype is blocked with 4-DAMP and the M_4_ subtype is blocked with tropicamide (TRO) or muscarínic toxin 3 (MT3). **(B)** shows the effect of neurotrophins and trophic cytokines (Brain-derived neurotrophic factor, BDNF, NT4, NT3, GDNF and CNTF) and related substances (TrkB-Fc chimera and k-252a). **(C)** shows the effect of voltage-dependent calcium channels (VDCC) blockers (nitrendipine [L type], ω-AGA [P type] and ω-CON [N type]), ion concentration change (0.5 mM magnesium) and PKC blockers (calphostin C (CaC) and chelerythrine (Che)). In all cases, significance is at *P* < 0.05.

The diagram in Figure 2A shows the effect of several subtype-selective muscarinic substances on ACh release in developing (P7; *M, S, W*) and mature (P30; *A*) nerve endings. The effect of the muscarinic substances on the PI of these treated muscles can be seen in R and the effect on the axonal loss rate can be seen in the outermost concentric layer.

In the adult (*A* contacts), M_1_ and M_2_ receptors modulate “evoked transmitter release by positive and negative feedback, respectively (Slutsky et al., 1999; Minic et al., 2002; Santafé et al., 2003, 2006). The M_2_ receptor inhibits ACh release because its selective block with methoctramine (MET) or AFX-116 increases release whereas the M_1_ receptor increases release because its selective block with pirenzepine (PIR) or MT-7 reduces it. Both M_1_- and M_2_-mediated mechanisms operate in parallel” (Tomàs et al., 2014), with some predominance of M_2_, because their simultaneous non-specific stimulation (oxotremorine, OXO) decreases release and the non-specific block (atropine, AT) increases transmitter output (data not represented in the figure, see Santafé et al., 2003). The M_3_ (4-DAMP) and M_4_ (tropicamide [TRO] and MT-3) blockers do not affect evoked ACh release. Thus, in the adult, mAChR signaling seems to “save the synapse function by decreasing the extent of evoked release” (Santafé et al., 2015) in basal conditions. Changes in synaptic activity may lead to subtypes playing different funcional roles (Minic et al., 2002; Santafé et al., 2003, 2006).

During developmental synapse elimination, the involvement of mAChR in ACh release is different. At P6-P7 roughly half of the NMJs are monoinnervated because one nerve terminal wins the axonal competition (Lanuza et al., 2002; Santafé et al., 2002). In these axons (*M* contacts), all the selective M_1_ and M_2_ blockers tested reduce release. Notably, the same occurs in the strongest endings of the dual junctions still in competition (*S* contacts). This suggests that a positive value of the winning axons is that all functional mAChR are committed to improve ACh release (in *M* and *S* contacts, the M_3_ and M_4_ blockers do not affect release). Using this autocrine mechanism the strongest ending may reinforce itself. However, in the weakest nerve contact in dual junctions (*W* contacts), only the M_2_ blockers reduce release whereas M_1_ and M_4_ blockers can lead to increases in the EPP evoked by these weak axons (Santafé et al., 2003, 2004, 2007b, 2009a,b; Tomàs et al., 2011). Thus, during NMJ synaptogenesis, the functional significance of the subtypes differs from the adult’s. M_2_ receptors promote release in all nerve endings independently of their ACh release level or maturation state whereas M_1_ and M_4_ receptors reduce release in the weakest endings on dual junctions. This suggests that the weak, presumably loser axon may be negatively influenced by ACh release from the strongest axons through M_1_ and M_4_ subtype pathways.

### Role of mAChR in the Recovery of Silent Synapses

In electrophysiological experiments, an increase in PI indicates the rapid recruitment of some silent synaptic contacts (*R* endings) that transitorily recover transmission. In P6-P7 muscles, we observed that blocking M_2_ with MET results in a percentage increase in the NMJ with 3–4 inputs and higher PI (Figure 2A). This was not the case for MT-7 (M_1_ blocker) or MT3 (M_4_ blocker). Thus, M_2_ seems to play a role in the recovery of silent synapses and might be involved in promoting the last step of the functional axonal disconnection (Tomàs et al., 2011). Whereas M_2_ may stimulate release in *M*, *S* and *W* axons, it seems to reduce it in silent endings because blocking M_2_ (MET) increases the ACh release in these endings just to become functionally recovered.

### mAChR in Axonal Loss

**Figure 3:**
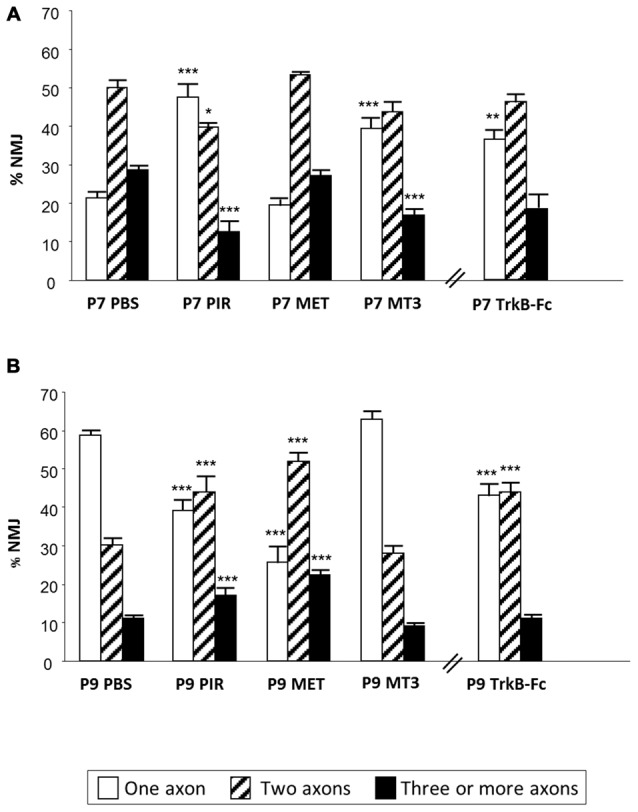
**Changes in polyneuronal innervation of the NMJ after inhibiting the mAChR and blocking the TrKB signaling.** The figure shows the percentage of singly-, doubly- and triply (or more) innervated NMJs in untreated YFP control mice (exposed to PBS applications) and after two (P7 in **A**) and four (P9 in **B**) applications (one application each day after P5) of the mAChR antagonists PIR, MET and MT3. The figure also shows the effect of the TrkB blocking pathway agent TrkB-Fc. ***p < 0.001,**p < 0.01, *p < 0.05. This figure has been adapted and redrawn from the Figures 3, 4 in the original article “[Presynaptic muscarinic ACh autoreceptors (M1, M2 and M4 subtypes), adenosine receptors (A_1_ and A^2A^) and tropomyosin-related kinase B receptor (TrkB) modulate the developmental synapse elimination process at the NMJ]” by Nadal et al. (2016). The original article is an open access article distributed under the terms of the Creative Commons Attribution License http://creativecommons.org/licenses/by/2.0, which permits unrestricted use, distribution and reproduction in any medium, provided the original work is properly cited.

By counting axons in P6-P7 YFP mice “we observed that M_1_ and M_4_ mAChR subtypes are involved in a mechanism that delays axonal elimination” (Nadal et al., 2016) because when M_1_ or M_4_ receptors are selectively blocked (with PIR and MT3 respectively), axonal loss is accelerated and causes a fast three-to-one axon transition (see the most external concentric layer in Figures 2A, 3). Interestingly, M_2_ does not change axonal loss rate in this period.

However, when we analyzed the effect of muscarinic agents at P9, we observed that the inhibitors PIR and MET (but not MT3) delay axonal loss (Figure 3, Nadal et al., 2016). Thus, “the M_1_-M_2_ subtype pair (in substitution of the M_1_-M_4_ pair) cooperates in favoring the full sequence of synapse elimination (the three-to-one axon transition)” (Nadal et al., 2016). Thus, mAChRs seem to play an important role in NMJ maturation and may affect ACh release capacity and the competitive strength of the different axons. Interestingly, even with the continued presence of the M_1_ and M_2_ inhibitors (PIR and MET, which delay axonal loss at P9), the axonal loss process comes to its normal end by the second postnatal week (P15; Nadal et al., 2016). This further suggests that other signaling pathways between the nerve terminals in competition cooperate to resolve the correct synaptic connectivity in a multifactorial process.

### Relation between mAChR-Mediated Changes in Axonal Loss and ACh Release

How are mAChR subtypes related to the ACh release ability of the *S* and *W* endings in polyinnervated synapses and the final loss of some axons? At P7, the ACh release capacity of the *W* endings (those that produce the smallest EPP) in dual junctions was increased by the M_1_ and M_4_ selective inhibitors PIR and MT3, whereas ACh release from the *S* nerve terminal was reduced (by PIR) or unaffected (by MT3; Santafé et al., 2003). Thus, these interferences reduce the difference in ACh release between *S* and *W* nerve endings in competition. This may mean a reduction in the competitive balance between these nerve terminals in terms of ACh release and a delay in axon elimination may be hypothetically expected. However, both PIR and MT3 accelerate axon loss at P7 and how this is related to the presumed lesser activity-related competition is not clear. A plausible interpretation is that in this developmental stage (P7), mAChR-mediated competition is fully operative in the NMJ of untreated muscles, and some axons, engaged in competition, have not been fully lost. If competition is reduced or unbalanced by, for instance, blocking M_1_ or M_4_ the loss of these axons accelerates. Also, in dual junctions ACh release is reduced by the M_2_ blocker MET in both the weak and strong endings suggesting that the axonal difference in release is the same but axons are not as strong or have less competitive force. In this case, as may be expected, MET does not affect axonal elimination at P7.

Between P7–P9, the percentage of multiinnervated NMJ changes only by a 10% and all NMJs are not finally monoinnervated until P15 (Lanuza et al., 2001, 2002; Santafé et al., 2001; Nelson et al., 2003). Also, the mAChR in the monoinnervated synapses does not mature functionally until P15 (Santafé et al., 2003) suggesting that the competitive interactions between axons and their release capacity may not be very different between P7 and P9. If this is so, “the reduction (at P9) in the competitive advantage (or disadvantage) linked to ACh release between the strong and weak endings produced by PIR and the reduccion of the strength of the different axons produced by MET” (Nadal et al., 2016), may result in a relevant delay in axonal loss and we found that this is the case. Thus, the relation between the ACh release capacity of the endings in competition and the rate of axonal loss in multiinnervated junctions seems best observed at P9 when, judging by the effects of PIR and MET, the receptors M_1_ and M_2_ play a role in accelerating axonal loss. The functional effect on ACh release of these receptors may reinforce the strongest endings and be detrimental to the weak endings in dual junctions.

## Neurotrophin Signaling

The agents that modify the mAChR response can alter the time course of the axons loss process but not the end point at P15 (Nadal et al., 2016). Likewise, experimental manipulations of the PKC/PKA intracellular pathways (for instance, blocking or stimulating PKC with Calphostin C (CaC) or phorbol esters, respectively) also change the time course but not the final synapse loss around P15 (Lanuza et al., 2002; Nelson et al., 2003), indicating that several receptors and their coupled intracellular mechanisms can be used for redundant synapse elimination (Nadal et al., 2016, 2017a,b).

### Neurotrophin Receptors in NMJ

Neurotrophins and their receptors have been shown to be expressed in muscle and nerve tissues both during development and in the adult (Funakoshi et al., 1993, 1995; Griesbeck et al., 1995; Gonzalez et al., 1999; Ip et al., 2001; Nagano and Suzuki, 2003; Pitts et al., 2006; Garcia et al., 2010c). Electrophysiology procedures show that some of these receptors influence ACh release in the NMJ in the same time periods (Stoop and Poo, 1996; Poo et al., 1999; Poo, 2001; Garcia et al., 2010b, d; Santafé et al., 2014).

### Neurotrophin Receptors in ACh Release

In the NMJ of adult rodents, exogenously added BDNF (or neurotrophin-4, NT-4) increases evoked ACh release after 3 h (Mantilla et al., 2004; Garcia et al., 2010d). This presynaptic effect can be prevented by preincubation with TrkB-Fc chimera or by pharmacologically blocking TrkB signaling (k-252a or the blocker antibody 47TrkB). Low doses of BDNF quickly promote (within minutes) a TrkB-dependent potentiation of transmitter release at developing NMJs in *Xenopus laevis* in culture (Stoop and Poo, 1996; Poo et al., 1999; Poo, 2001). In P7 developing muscles *ex vivo* (Figure 2B), exogenous BDNF (10 nM for 3 h or 50 nM for 1 h) potentiates release in all endings also with the involvement of TrkB receptors (Garcia et al., 2010c). NT-3 potentiates release only in the *W* and *S* endings in dual junctions (Garcia et al., 2010d), and NT-4 only in adult NMJs. The Glial cell line-derived neurotrophic factor (GDNF) and Ciliary neurotrophic factor (CNTF), on the other hand, do not directly modify ACh release in any nerve endings (Garcia et al., 2010a).

Thus, “exogenous BDNF acts on a section of the release mechanism that is operative and potentiates neurotransmission in all nerve endings” (Garcia et al., 2010b) that are in developmental competition (regardless of their particular state of maturation). However, when we analyzed the possible effect of endogenously produced BDNF during synaptic maturation, we found that blocking TrkB (k-252a) or neutralizing endogenous BDNF (TrkB-Fc) does not change the quantal content of the *W* endings although surprisingly it does increase release in the S endings (Garcia et al., 2010d; see Figure 2B). Therefore, although the BDNF-TrkB pathway seems ready to be stimulated by exogenous BDNF to potentiate release in all nerve terminals during development, endogenous BDNF does not affect the weak ending at P7 but, in this period, may help to reduce release in the *S* nerve terminal (Garcia et al., 2010d). The effect of BDNF on *S* endings may be related to the relative involvement and generally opposing actions of truncated and full-length TrkB and p75^NTR^ receptors, and proBDNF and mature BDNF on the postnatal polyinnervated synapses.

### Neurotrophin Receptors in Silent Synapses. Role of TrkB in Recovery of Silent Synapses

Blocking TrkB, using TrkB-Fc to prevent endogenous BDNF action or stimulating with several neurotrophins (NT-4, NT-3, GDNF or CNTF) does not change mean PI. However, stimulation with exogenous BDNF (1 h in the bath) transitorily increases PI, considerably reduces monoinnervated junctions and increases the number of junctions with 2–3 functional inputs (Tomàs et al., 2011); the innermost concentric layer in Figure 2B). This suggests that there are a number of silent inputs on the boundary that can be recovered (to produce an EPP) by BDNF. In fact, BDNF stabilizes silent synapses at mice NMJs during development (Kwon and Gurney, 1996; Garcia et al., 2010d). It can be hypothesized that the lack of activity in the weakest endings means that little BDNF is produced and it does not work locally on these endings (the absence of tonic change in ACh release by TrkB block or by using the neutralizing fusion protein TrkB-Fc suggests that endogenous BDNF does not work on the weak and silent synapses). However, exogenous BDNF may reach the weak endings close to elimination and induce some release recovery. As previously stated, downregulation of M_2_ (MET) produces the same effect as TrkB stimulation with exogenous BDNF (that is to say, PI increases because silent endings recover some of their transmitter release capacity). ACh from the strong more active terminals may reach M_2_ in the neighbor silent endings thus punishing them.

### Neurotrophin Receptors in Developmental Axonal Loss

We used TrkB-Fc to sequester endogenous BDNF and NT-4 and, at P7, morphologically observed a clear acceleration of the three-to-two rate in axon loss, well matched by the acceleration of the two-to-one rate (see the outermost concentric layer in Figures 2B, 3, and also Nadal et al., 2016) although, as stated above, the funcional PI does not change significantly. This seems to suggest that some of the axonal endings eliminated are not functional at this time (the opposite of what occurs with MET—see above—which has no effect on the number of axons at P7 but increases the percentage of functional ones). Interestingly, using a chemical-genetic approach to block TrkB signaling during NMJ development, it has been found that “inhibition of TrkB signaling by daily injection of 1NMPP1 to TrkBF616A knock-in mice accelerated synapse elimination at P7” (Je et al., 2013). This fastened synapse elimination is similar to the described here in developing NMJ at P7. Therefore, in normal conditions the physiological role of the BDNF-TrkB pathway at P7 seems to delay the axonal loss process although endogenous BDNF does not affect ACh release in the W endings, as stated above, and transmitter release seems to be somewhat independent. This result partially agrees with a proposed model in which proBDNF and mature BDNF (mBDNF) serve as potencial “punishment” and “reward” signals for the less active and more active nerve endings, respectively *in vivo*. Exogenous proBDNF promoted synapse elimination by activating p75^NTR^ receptors, whereas mBDNF infusion substantially delayed synapse elimination in the mouse LAL muscle (Je et al., 2013). The postsynaptic secretion of “proBDNF stabilizes or eliminates presynaptic axon terminals, depending on its proteolytic conversion at synapses” (Je et al., 2013). “Pharmacological inhibition of the proteolytic conversion of proBDNF to mBDNF accelerated synapse elimination via activation of p75^NTR^ receptors. Furthermore, the inhibition of both p75^NTR^ receptors and sortilin signaling attenuated synapse elimination” (Je et al., 2013). “It seems that proBDNF-mediated synaptic retraction requires simultaneous activation of p75^NTR^ receptors and the complementary receptor sortilin, a coreceptor that binds to pro-neurotrophins” (Je et al., 2013; see also Nykjaer et al., 2004; Teng et al., 2005; Jansen et al., 2007). Also, in the LAL muscle of the mouse, blocking the p75^NTR^ receptors delays axonal loss and some nerve terminals even regrow (Garcia et al., 2011).

However, “at P9, neurotrophin signaling seems to reverse their coupling to the axonal loss process (Figure 3) because TrkB-Fc considerably delays elimination (resulting in more dual and fewer monoinnervated NMJs), which indicates that in a normal situation the role of BDNF/NT-4 mediators changes at this time (P9) and accelerates elimination, as has been described above for the muscarinic mechanism” (Nadal et al., 2016). In PC, the deficiency of TrkB has a consequence in the developmental detach and loss of redundant CF synaptic contacts. It can be observed “an abnormal multiple CF innervation in PC in trkB*-*deficient mice” Bosman et al., 2006) in the second postnatal week (see also Watanabe and Kano, 2010). This delay in synapse elimination is similar to the described by us in developing NMJ (at P9) treated with TrkB-Fc.

Thus, also in this case, it seems that the BDNF-TrkB pathway plays a biphasic role during the critical period of synapse loss. The progressive maturation of the NMJ at P9 may change the operating conditions of the BDNF-TrkB pathway to a more mature endogenous BDNF production and release promoting effect in certain nerve terminals resulting in more efficient competitive interactions and axonal loss.

## Relation Between Muscarinic and Neurotrophin Signaling

“Synapse operation is largely the logical outcome of the confluence of several metabotropic receptors and signaling” (Tomàs et al., 2014). In the adult NMJ, “the activity of a given receptor can modulate a given combination of spontaneous, evoked and activity-dependent ACh release parameters" (Tomàs et al., 2014). Specifically, the mAChR generally seems to protect the synapse from resources depletion by decreasing the extent of evoked ACh secretion (mainly an M_2_ action) and decreasing activity-dependent depression (Santafé et al., 2003). One of the main roles of TrkB is to keep the spontaneous quantal leak of ACh low and potentiate evoked release (Garcia et al., 2010d). Thus, some functions in the adult synapses can be balanced by the opposing actions of different receptors.

Changes in how some of these receptors and pathways operate affect the normal coupling of the other complementary molecules to transmitter release. “Consecutive incubations with two substances (for instance, a muscarinic blocker followed by a TrkB blocker) can be used as a pharmacological tool to investigate the possible occlusive or additive crosstalk effects between two receptors” (Tomàs et al., 2014). In the adult NMJ, we found a link between mAChR and TrkB pathways because the normal function of the mAChR is a requirement for the TrkB to couple to ACh release and vice versa (Garcia et al., 2010d; Santafé et al., 2014). It is know that mAChR and TrkB pathways are related and share a link mediated by phospholipase C (PLC)-phosphatidylinositol 4,5-bisphosphate (PIP2)-diacylglycerol (DAG)-PKC, which modulates P/Q-type VDCC (Santafé et al., 2006; Amaral and Pozzo-Miller, 2012). Also, “the PLC-generated DAG regulates the vesicle priming protein Munc13–1 and recruits ACh-containing vesicles for the immediately releasable pool” (see Bauer et al., 2007; Tomàs et al., 2014). Thus, the relations between these signaling pathways contribute to modulate the VDCC and synaptic vesicles, and then neurotransmission (Takamori, 2012). The inflow of Ca^2+^ needed for ACh release is modulated by the presynaptic M_1_ mAChR (Santafé et al., 2006) interacting with the BDNF-TrkB pathway (Amaral and Pozzo-Miller, 2012). In the adult skeletal NMJ, the M_1_ mAChR contribute to adjust the M_2_ mAChR subtype, which is a protein kinase A (PKA)-mediated inhibitor of ACh secretion (Santafé et al., 2006). This balance is further adjusted by adenosine coreleased with ACh at the NMJ (Oliveira et al., 2009; Garcia et al., 2013; Santafé et al., 2015) and TrkB (Garcia et al., 2010c). However, when neuromuscular transmission is low (as it is during synaptic development) or defective, “the balance between them shifts in favor of the M_1_ mAChR, partly because of an M_2_ mAChR-mediated switch from PKA to PKC activation” (see Santafé et al., 2007a; Garcia et al., 2010b; Tomàs et al., 2014).

The complementary function of these receptors in the adult NMJ neurotransmission provides further evidence of their coordinated involvement in developmental synaptic elimination. Thus, PKC, and VDCC can also be expected to have a role in developmental axonal loss. PKC is not coupled to modify transmitter release in basal conditions because its inhibition with, for instance, CaC does not influence the quantal content of the EPP. This is the situation in adult motor nerve endings, and also in the strong endings of dually innervated NMJ and in the ending in the recently monoinnervated junctions during maturation (Santafé et al., 2007a, 2008); see also Figure 2C). In these nerve terminals, however, PKC couples to potentiate ACh release during synaptic activity (Santafé et al., 2007a). Interestingly however, a tonic PKC coupling reduces release in the weakest axons in dual junctions because their inhibition (with CaC or chelerythrine, Che) increases ACh release in these endings and even recovers *R* endings (Figure 2C). Therefore, PKC may be decisive for the axonal loss control (Lanuza et al., 2002; Nelson et al., 2003; Santafé et al., 2009a). As far as the VDCC and calcium inflow are concerned, our results show (judging by the effect of the inhibitors used, Figure 2C) that a part of the calcium entry through the P/Q-, N- or L-type VDCC reduces ACh release in the weak endings of dual junctions (Santafé et al., 2009a) and even also recover *R* endings. Therefore, our results indicate that during NMJ development “there is a release inhibition mechanism based on a mAChR-PKC-VDCC intracellular cascade. When it is fully active in certain weak motor axons, it can depress ACh release and even disconnect synapses” (Santafé et al., 2009a). Blocking PKCs, VDCCs (P/Q-type with ω-Agatoxin [ω-AGA], N-type with ω-Conotoxin [ω-CON] or L-type with nitrendipine [NIT]) and/or calcium influx (moderately increasing Mg^2+^) or mAChR (M_1_- and/or M_4_-subtypes) “can lead to similar percentage increases in the size of the synaptic potentials evoked by weak axons in developing polyinnervated synapses and even, in most cases, the functional recovery of previously disconnected synapses” (Santafé et al., 2009a; see also Santafé et al. 2003; 2004; 2007b; 2009b; Tomàs et al., 2011). “We suggest that this mechanism plays a central role in the elimination of redundant neonatal synapses” (Lanuza et al., 2014). However, at P7, M_1_, M_4_ and TrkB receptors delay axon loss while M_1_ and M_4_ reduce ACh release in the weakest axon terminals, which suggests some independence between transmitter release and elimination. We interpret that at this developmental point the activity-dependent competitive interactions in most junctions are at their peak and this may delay axon loss. The accelerating effect of these receptors on axon loss is much clearer at P9.

Cerebellar CF to PC synapses in rodents provides “a good model to study elimination of redundant synapses in the central nervous system. At birth, each PC is innervated by multiple CFs but at the end of the third postnatal week, most PCs become innervated by single CFs” (Hashimoto and Kano, 2005; see also Kano and Hashimoto, 2009). In the early phase of CF synapse elimination, CF differential activities results in Ca^2+^ influx through the P/Q- type VDCC in an activity-dependent manner. This promotes competition among CF inputs allowing the strongest CF to segregate in the dendrites, whereas the weaker fibers “remain on the soma until their perisomatic synapses are massively eliminated” (Watanabe and Kano, 2010). A signaling cascade from a glutamate receptor (mGluR1) to PKCγ is involved in this late phase of CF synapse loss. Mutant mice deficient at some point in the pathway show reduced CF synaptic loss (Kano et al., 1995, 1997, 1998; Levenes et al., 1997; Offermanns et al., 1997; Hashimoto et al., 2000, 2001; Ichise et al., 2000). Interestingly, mice deficient in “P/Q-type Ca^2+^ channel have persistent multiple CF innervation on the PC soma” (Miyazaki et al., 2004). This fact agrees with our observation that, in the NMJ, a fraction of the Ca^2+^ entry through the P/Q-, N- or L-type VDCC reduces ACh release in the weak endings of dual junctions (Santafé et al., 2009a) relating Ca^2+^ inflow, transmitter release and synapse loss.

Interestingly, mAChR (Garcia et al., 2005) and TrkB receptors (Garcia et al., 2010d) are present also in the postsynaptic membrane contributing to their organization (Gonzalez et al., 1999; Belluardo et al., 2001; Loeb et al., 2002; Peng et al., 2003). In this postsynaptic membrane, selective nAChR-phosphorylation by PKC (in the delta subunit) and PKA (epsilon subunit) is a major cause of nAChR dispersion and stability, respectively (Nishizaki and Sumikawa, 1994; Li et al., 2004; Lanuza et al., 2010). PKC-induced dispersion under the weakest nerve terminals and a PKA-induced catching and stabilization under the more active axon terminals results in the differentiation of the postsynaptic gutters. In our blocking experiments of these receptors, we observed that prolonged M_1_, M_2_ and TrkB block produce a delay in postsynaptic maturation at P15. This indicates a role for these receptors in the postsynaptic component. Nevertheless, this occurs when axon loss has been completed, suggesting independent regulation (Nadal et al., 2016).

Interestingly, glutamate and mGluR1 also mediate transmission at the NMJ (Waerhaug and Ottersen, 1993; Malomouzh et al., 2011; Walder et al., 2013). Glutamate at the NMJ is derived from the motor nerve terminal (Marmiroli and Cavaletti, 2012). Postsynaptic NMDA receptors at the end plate have been documented in rodent myotubes (Lück et al., 2000). It has been found that developmental synapse loss in mice is slowed at P11 by reducing activation of the glutamate-NMDA receptor pathway (Personius et al., 2016). This is in accordance with our observation of the effect of mAChR and TrkB receptors block delaying axonal loss at P9 and postsynaptic gutters maturation around P15. The involvement of the mGluR1s in synapse elimination at the NMJ emphasize the complexity of a multireceptor mechanism.

## Conclusion

**Figure 4:**
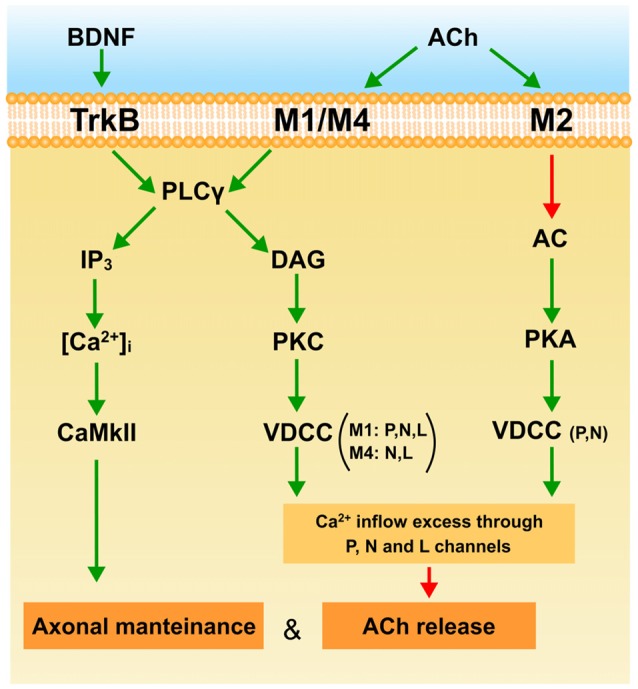
**The figure is a plausible interpretation of the role of mAChR (M_1_, M_2_ and M_4_) and the BDNF-TrkB pathways in the process of eliminating the weakest endings around P7.** The explanation can be found in the conclusion to the main text. Green arrows indicate activation or stimulation, and red arrows inhibition.

The diagram in Figure 4 shows a plausible interpretation of the role of mAChR and BDNF-TrkB pathways in the elimination of the supernumerary endings in the NMJ at the end of the first week postnatal (P7). ACh released from *W* and *S* axons in the common synaptic cleft could stimulate M_1_, M_4_ and M_2_ muscarinic types in *W* endings. M_1_ receptors reduce release through the PKC pathway due to an excess of Ca^2+^ inflow (through P, N and L VDCC) or because a selective inflow through L channel (only present in *W* endings) targeted to restrain the release machinery. The coupling of M_1_ to PKC activity in the *W* endings differs from the coupling of the mature and adult (*M* and *A*) synapses where release is potentiated using Ca^2+^ inflow through the P-channel. The presence of M_4_ and the L and N channels in the *W* contacts may have something to do with this difference. The PKA-linked M_2_ subtype is also present in the *W* axons. It is related only to P and N channels and here potentiates ACh release which, in this case also, differs from the adult where M_2_ inhibits release. Thus, the weak and presumably loser axon may be negatively influenced by their neighboring strongest one through M_1_ and M_4_ subtypes. The axons that win the competition (*M* and *S* contacts) have their functional mAChR (M_1_, M_2_) committed to improve ACh release. Using this autocrine mechanism the strongest ending may reinforce themselve. However, when axon loss is analyzed at P7, the M_1_-M_4_ pair reduces the synapse elimination rate. This effect may be produced by the IP3-CaMKII pathway which is known to promote axonal maintenance and growth. It seems that at P7, mAChRs contribute to the development of activity-dependent competition but that, at this time, these competitive interactions can delay axon loss.

At P7 the BDNF-TrkB pathway is operative and ready to be stimulated by exogenous BDNF to favor ACh release in all axonal endings during development (*W*, *S* and *M* endings and even transitorily recover some silent axons and increase PI). This effect may be produced by the IP3 branch of the PLC because endogenous BDNF does not affect ACh release in the weak ending. Interestingly, at P7 the BDNF-TrkB pathway delays axonal loss in the same way as mAChR signaling does. This effect may also be mediated by the CaMKII pathway.

Thus at P7, TrkB, M_1_ and M_4_ promote axonal maintenance. This coincides with mAChR reducing ACh release in the weakest axon in dual NMJs, which may result in their being competitively handicapped. However, some days later at P9, the mAChR subtype pair M_1_-M_2_ and the BDNF pathway cooperate to favor the full sequence of axonal loss and synapse elimination.

## Author Contributions

LN, EH, AS, VC and MT: data collection, quantitative analysis; literature search, data interpretation, design graphic; NG, MAL and MMS: statistics; JT, NG, MAL and MMS: conception and design, literature search, data interpretation, manuscript preparation.

## Ethics Statement

The mice were cared for in accordance with the guidelines of the European Community’s Council Directive of 24 November 1986 (86/609/EEC) for the humane treatment of laboratory animals. All experiments on animals have been reviewed and approved by the Animal Research Committee of the Universitat Rovira i Virgili (Reference number: 0233).

## Conflict of Interest Statement

The authors declare that the research was conducted in the absence of any commercial or financial relationships that could be construed as a potential conflict of interest.

## Abbreviations

ACh: acetylcholine
AR: adenosine receptors
AT: atropine
BDNF: Brain-derived neurotrophic factor
CaC: calphostin C
Che: chelerythrine
CF: climbing fiber
CNTF: Ciliary neurotrophic factor
DAG: diacylglycerol
EPP: evoke endplate potentials
GDNF: Glial cell line-derived neurotrophic factor
mGluR1: glutamate receptor
LAL: Levator auris longus muscle
mAChR: muscarinic acetylcholine receptor
nAChR: nicotinic acetylcholine receptor
MT-3: muscarinic toxin 3
MT-7: muscarinic toxin 7
M_1_, M_1_: -type muscarinic acetylcholine receptor
M_2_, M_2_: -type muscarinic acetylcholine receptor
M_4_, M_4_: -type muscarinic acetylcholine receptor
MET: methoctramine
NIT: nitrendipine
NMJ: neuromuscular junction
NTR: neurotrophin receptors
NT-4: neurotrophin-4
OXO: oxotremorine
PIR: pirenzepine
PKA: protein kinase A
PKC: protein kinase C
PLC: phospholipase C
PC: Purkinje cells
TrkB: tropomyosin-related kinase B receptor
TRO: tropicamide
VDCC: voltage-dependent calcium channels
ω-AGA: ω-agatoxin
ω-CON: ω-conotoxin.
